# Supplementary material for: Determination of reference intervals for knee motor functions specific to patients undergoing knee arthroplasty
Source: PLoS One. 2021 Apr 14;16(4):e0249564. doi: 10.1371/journal.pone.0249564 (PMC8046200; doi:10.1371/journal.pone.0249564)

S1 Fig. Determination of reference intervals by parametric method

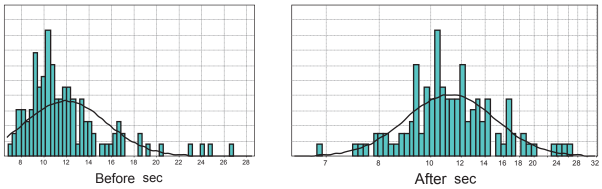

TUGcom(sec) MF n=118
Para: 7.59 ~ 11.22 ~ 20.57
Nonpara: 7.40 ~ 10.95 ~ 23.70
Pow=0.237 TPos=6.528
Kurt=0.095 Skew=0.338
Age ~69 K-S test P=0.635 (N.S.)

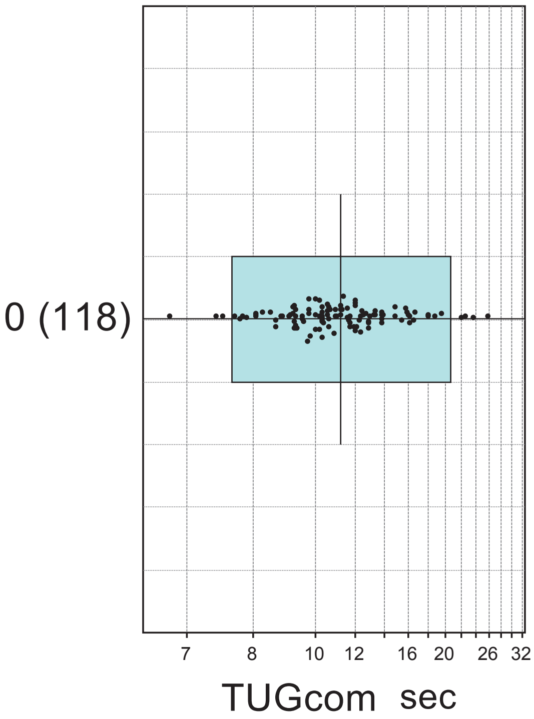

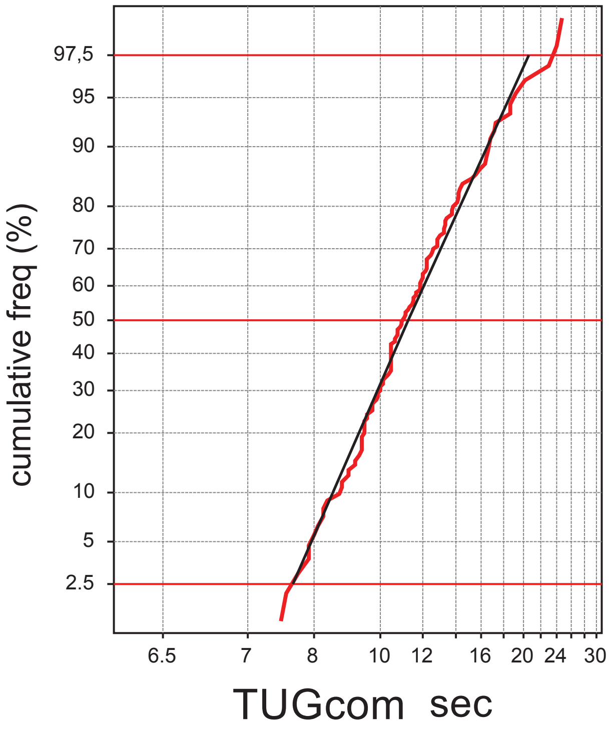

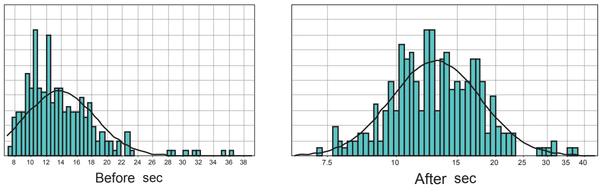

TUGcom(sec) MF n=281
Para: 8.01 ~ 12.86 ~ 24.85
Nonpara: 7.80 ~ 12.70 ~ 29.39
Pow=0.268 TPos=6.652
Kurt=0.063 Skew=0.046
Age 70~79 K-S test P=0.940 (N.S.)

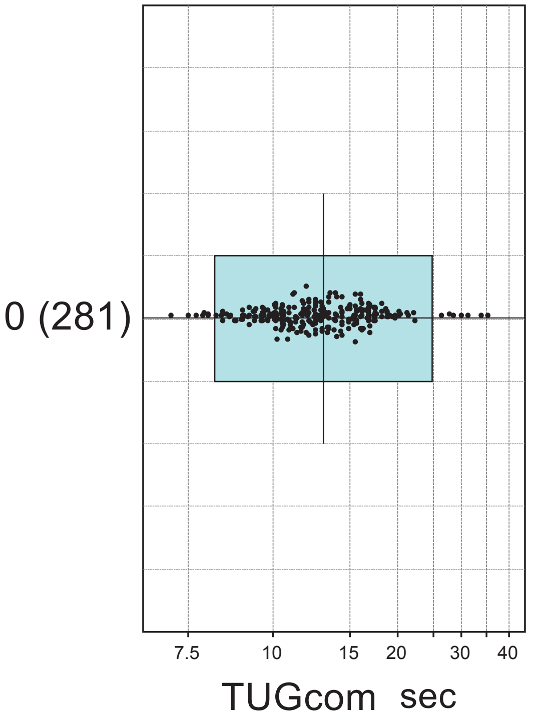

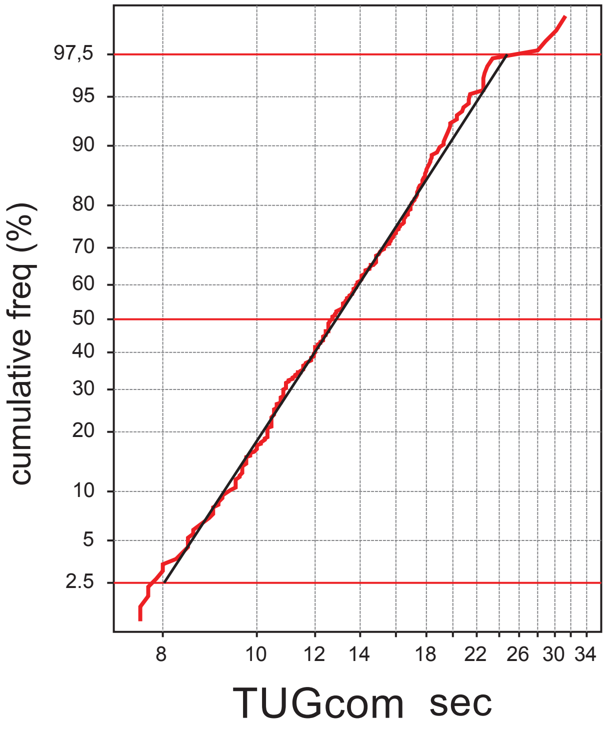

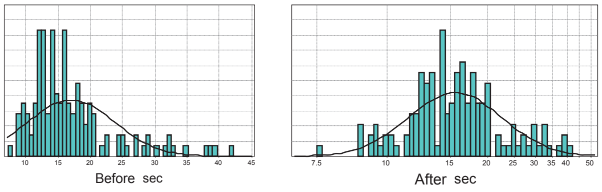

TUGcom(sec) MF n=140
Para: 8.90 ~ 15.55 ~ 33.55
Nonpara: 8.80 ~ 15.60 ~ 37.90
Pow=0.091 TPos=5.881
Kurt=-0.144 Skew=0.099
Age 80~ K-S test P=0.730 (N.S.)

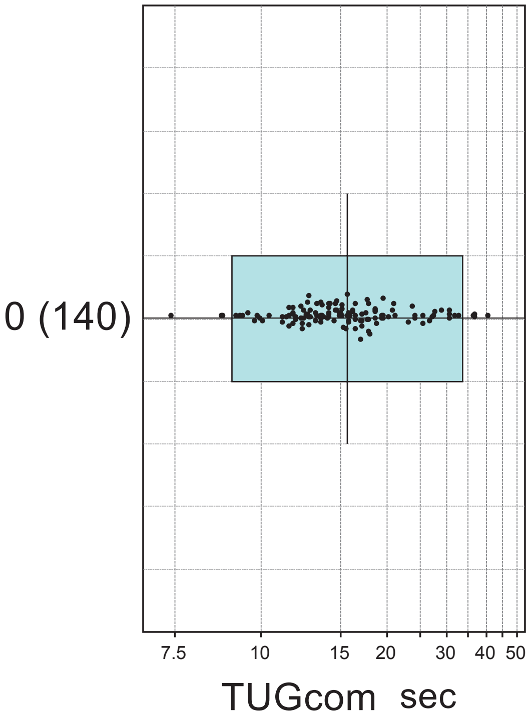

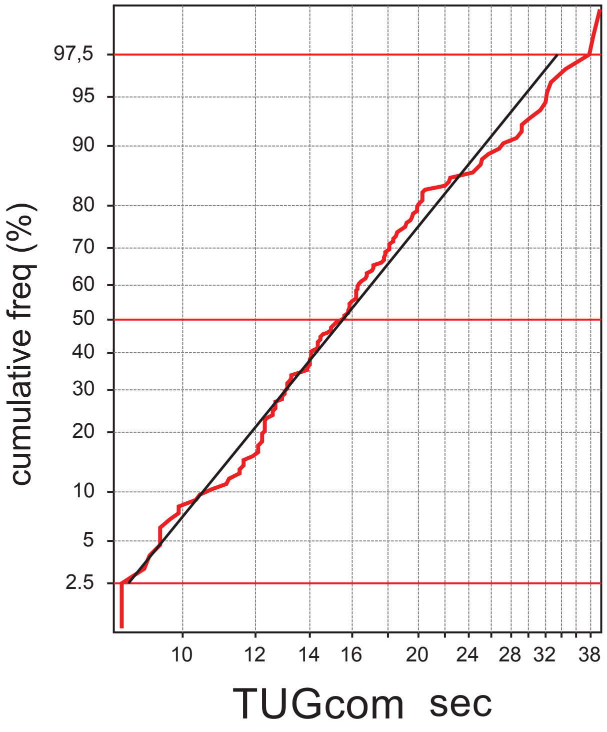

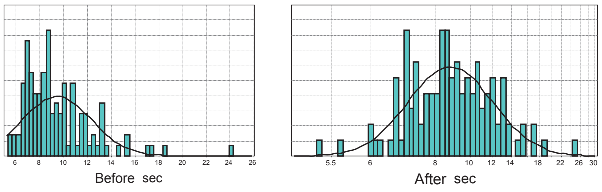

TUGmax(sec) MF n=118
Para: 5.93 ~ 8.73 ~ 16.98
Nonpara: 6.00 ~ 8.55 ~ 15.65
Pow=0.188 TPos=5.178
Kurt=-0.014 Skew=0.143
Age ~69 K-S test P=0.585 (N.S.)

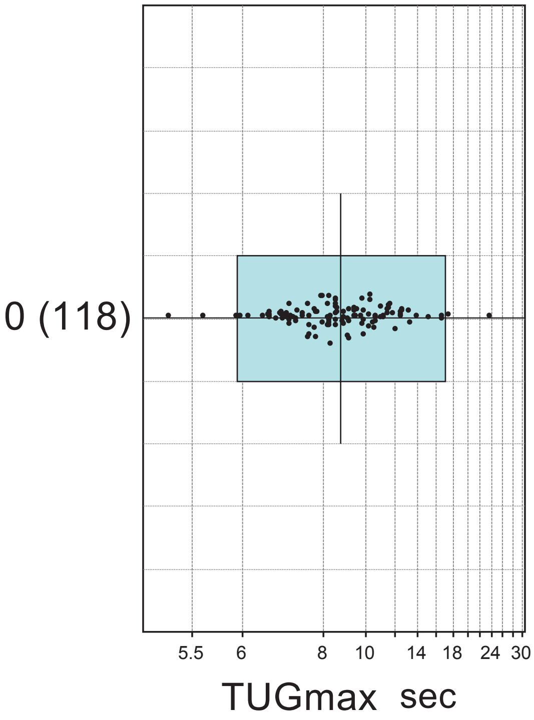

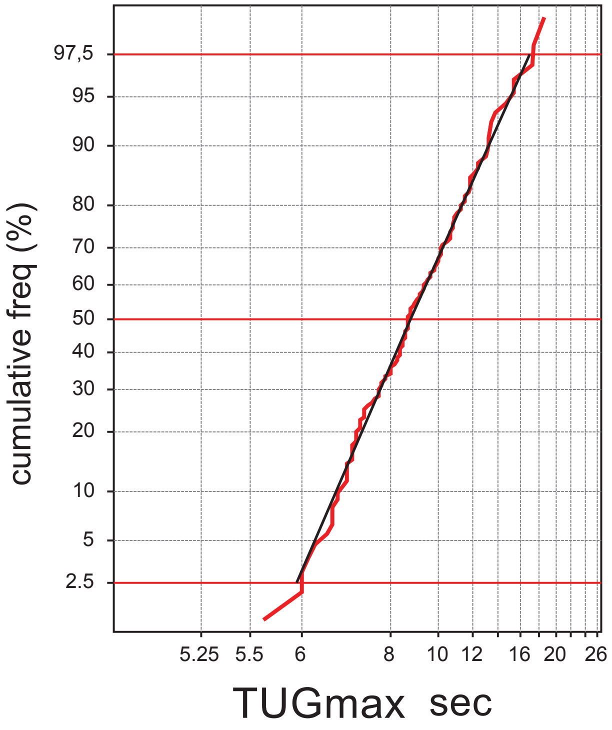

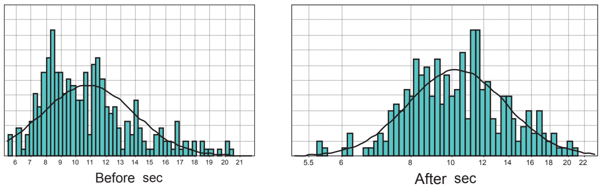

TUGmax(sec) MF n=279
Para: 6.41 ~ 10.30 ~ 18.29
Nonpara: 6.20 ~ 10.40 ~ 19.79
Pow=0.345 TPos=5.094
Kurt=-0.332 Skew=0.129
Age 70~79 K-S test P=0.191 (N.S.)

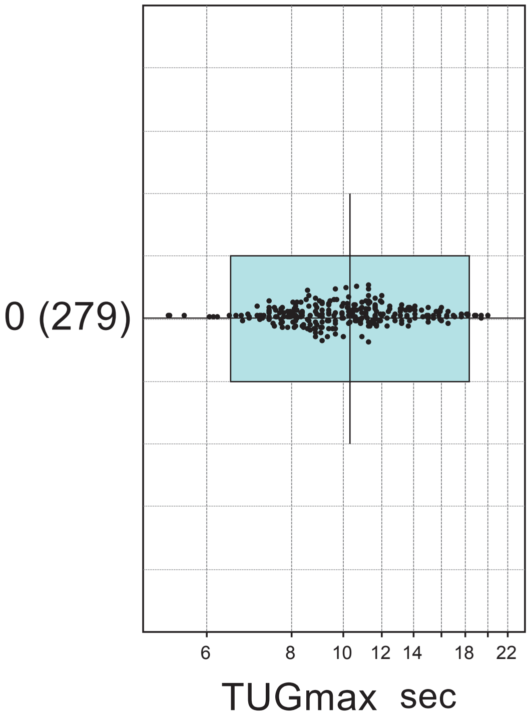

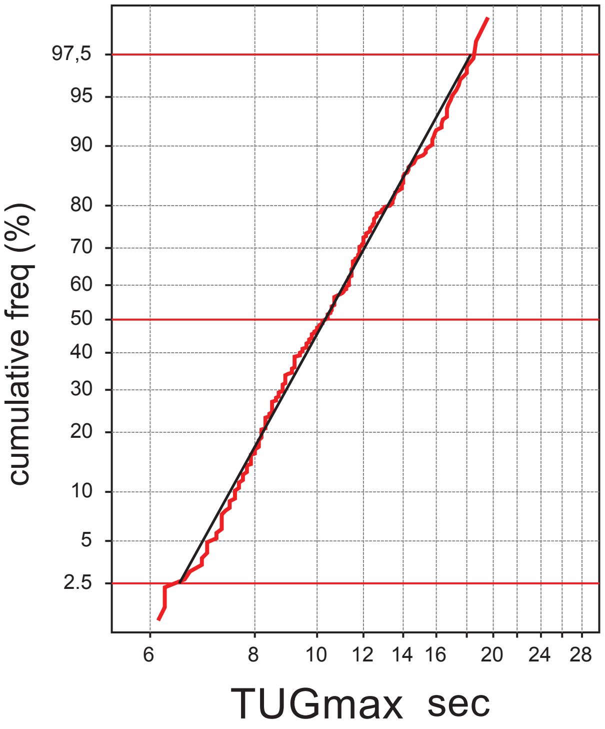

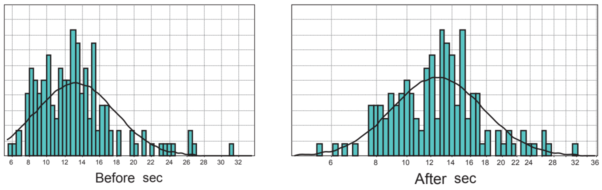

TUGmax(sec) MF n=138
Para: 6.69 ~ 12.62 ~ 23.43
Nonpara: 7.00 ~ 12.85 ~ 26.90
Pow=0.439 TPos=4.799
Kurt=-0.014 Skew=-0.048
Age 80~ K-S test P=0.810 (N.S.)

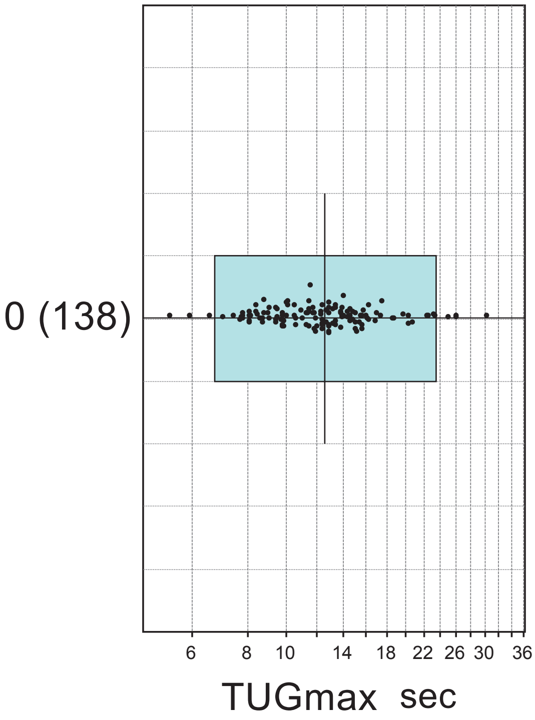

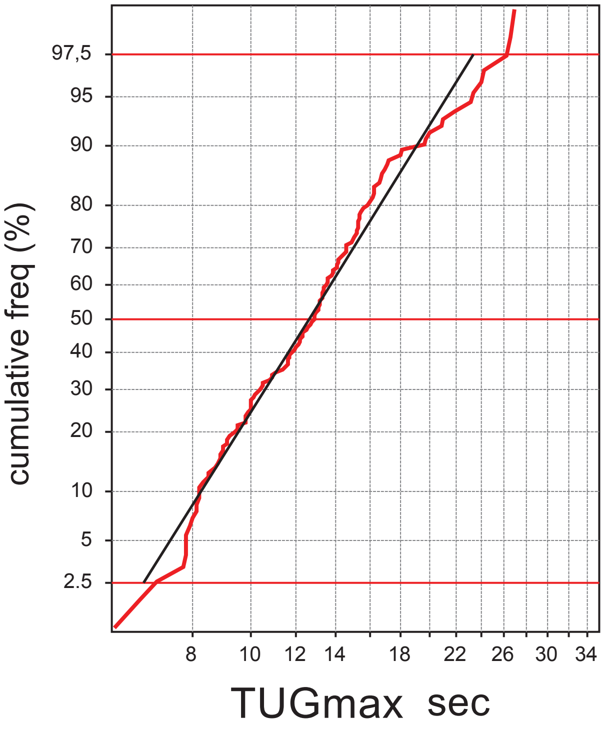

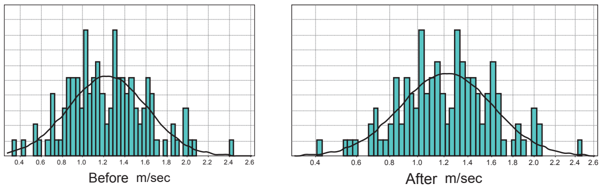

Maximum Walking Speed(m/sec) MF n=120
Para: 0.596 ~ 1.222 ~ 2.025
Nonpara: 0.540 ~ 1.220 ~ 2.020
Pow=0.711 TPos=0.337
Kurt=-0.449 Skew=0.02
Age ~69 K-S test P=0.420 (N.S.)

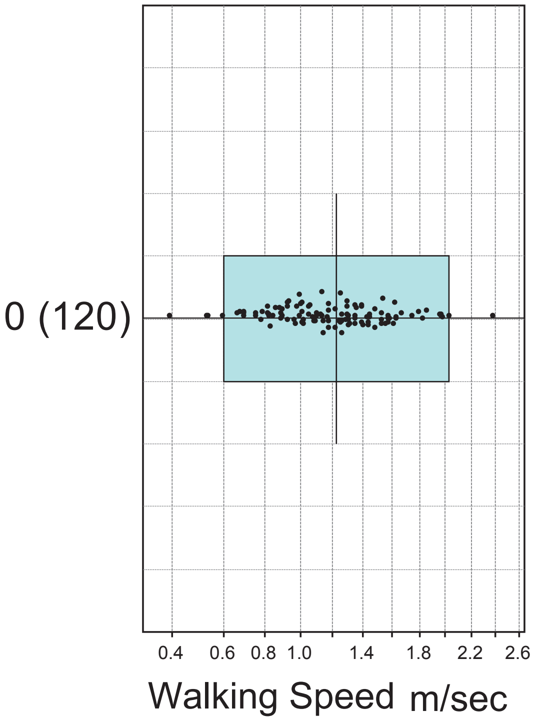

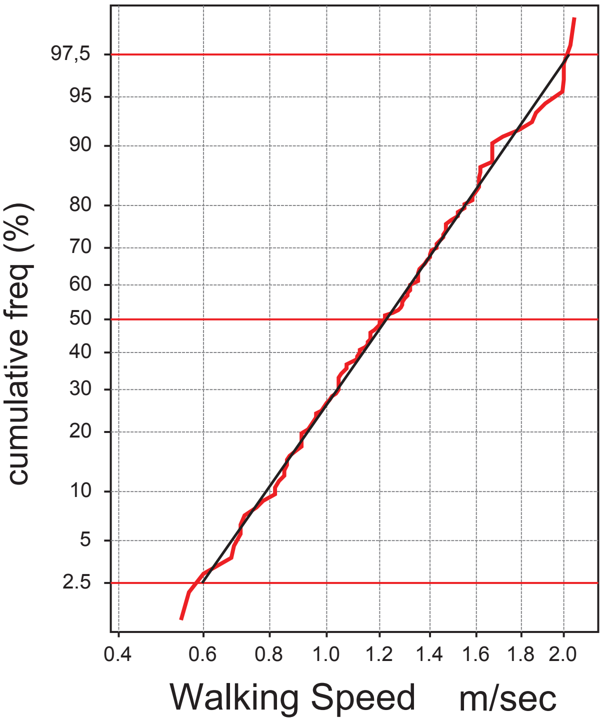

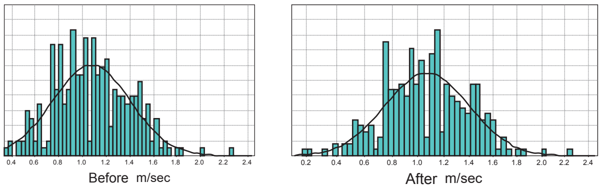

Maximum Walking Speed(m/sec) MF n=285
Para: 0.453 ~ 1.069 ~ 1.733
Nonpara: 0.500 ~ 1.060 ~ 1.678
Pow=0.898 TPos=0.112
Kurt=-0.482 Skew=0.028
Age 70~79 K-S test P=0.850 (N.S.)

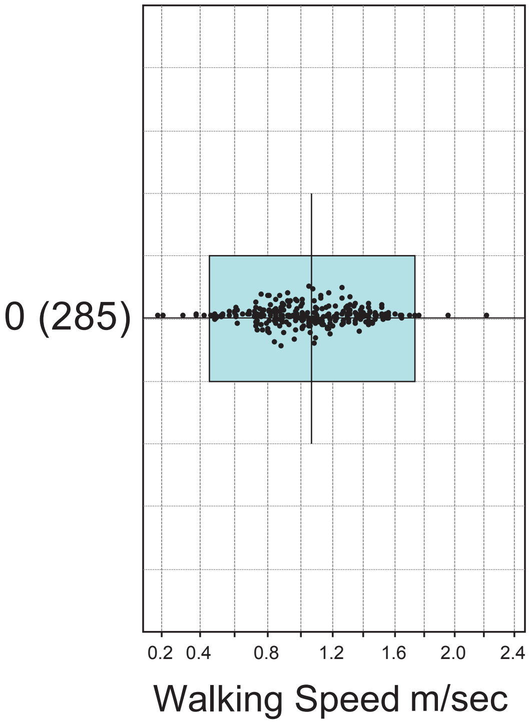

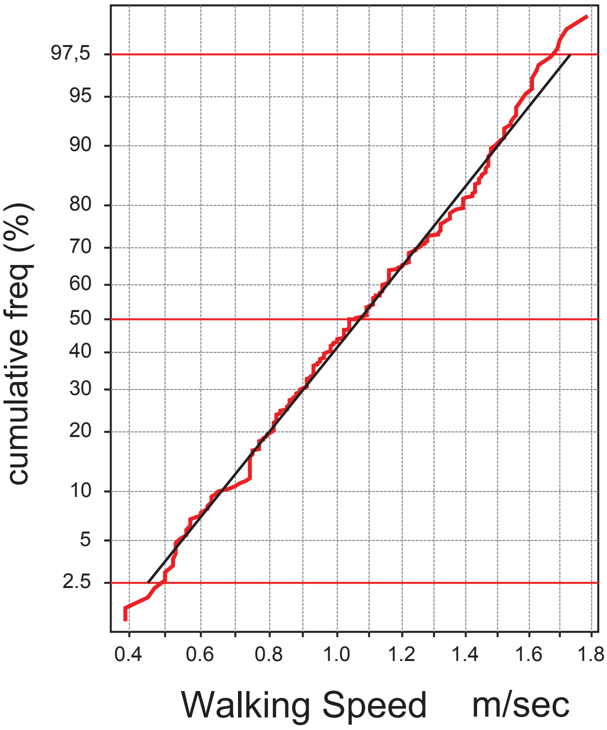

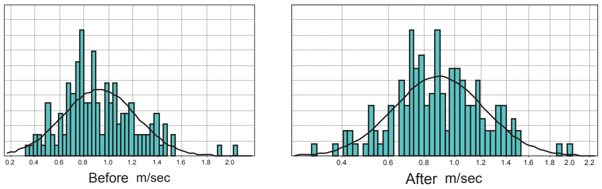

Maximum Walking Speed(m/sec) MF n=140
Para: 0.432 ~ 0.897 ~ 1.590
Nonpara: 0.410 ~ 0.875 ~ 1.520
Pow=0.534 TPos=0.205
Kurt=-0.32 Skew=0.122
Age 80~ K-S test P=0.856 (N.S.)

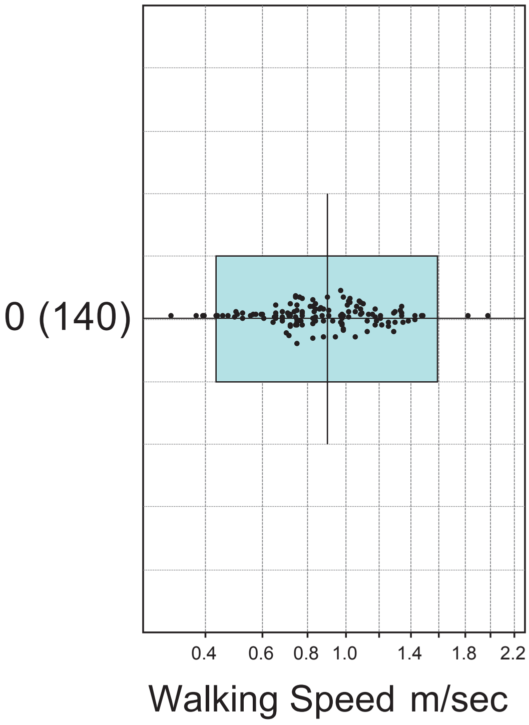

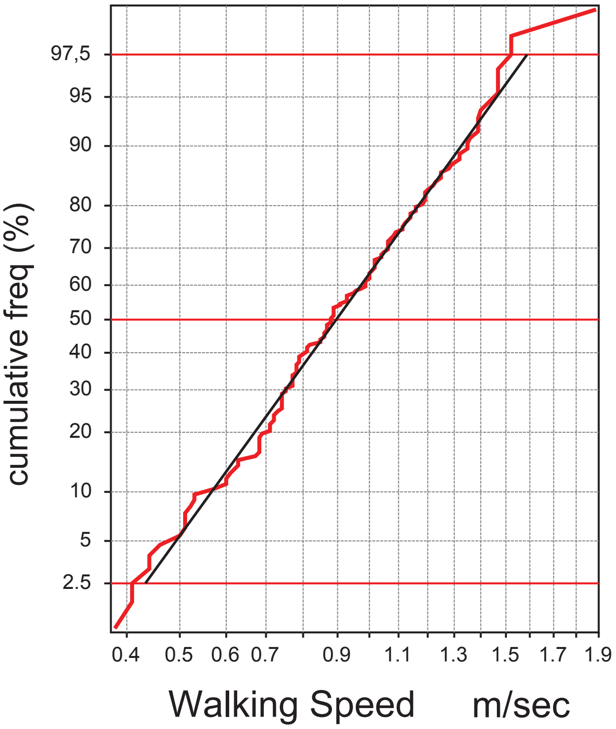

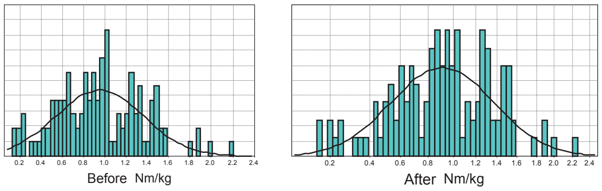

Extensor muscle strength(Nm/kg) Male n=124
Para: 0.253 ~ 0.914 ~ 1.876
Nonpara: 0.192 ~ 0.935 ~ 1.864
Pow=0.62 TPos=0.045
Kurt=-0.113 Skew=-0.208
K-S test P=1.000 (N.S.)

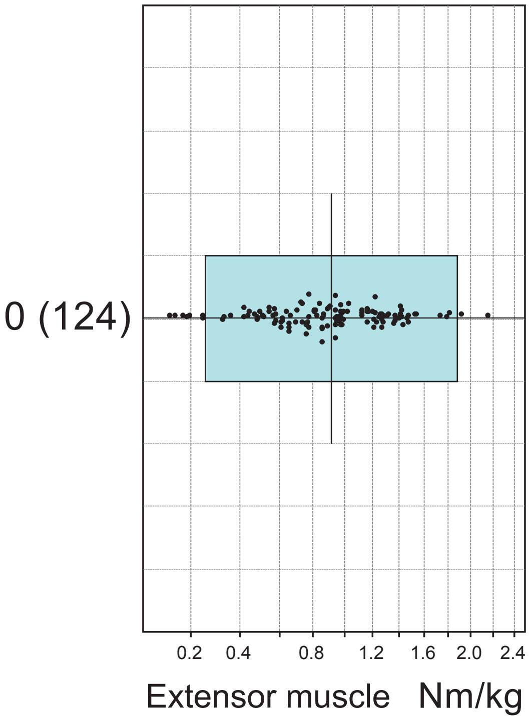

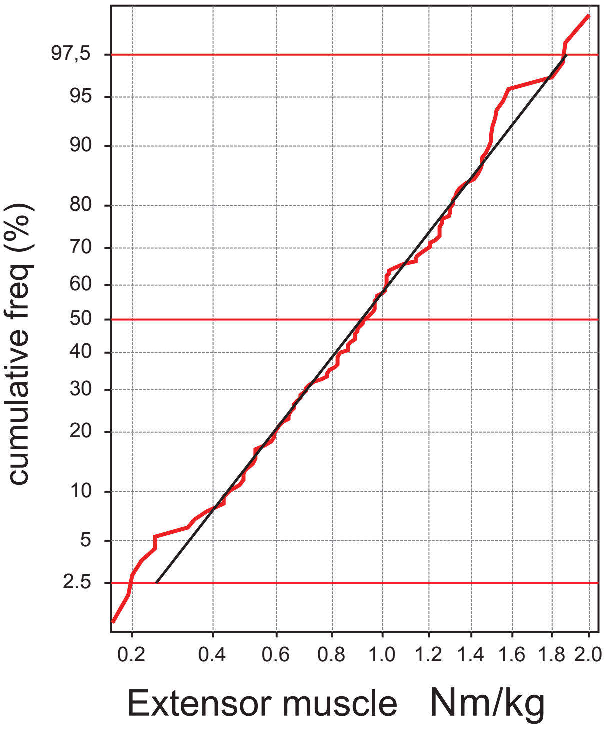

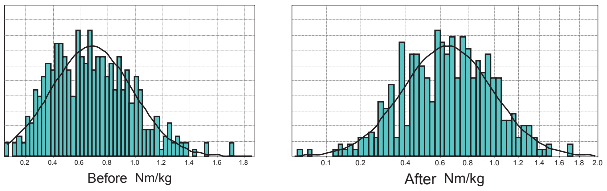

Extensor muscle strength(Nm/kg) Female n=404
Para: 0.196 ~ 0.651 ~ 1.358
Nonpara: 0.200 ~ 0.660 ~ 1.304
Pow=0.569 TPos=0.056
Kurt=-0.458 Skew=-0.104
K-S test P=0.993 (N.S.)

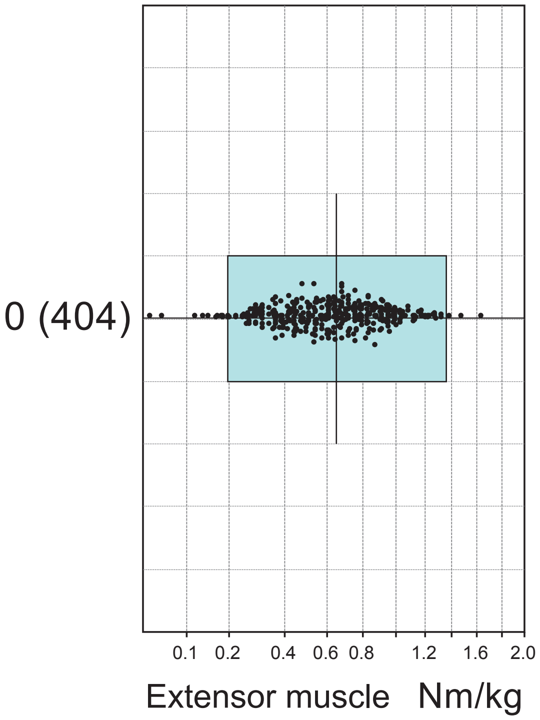

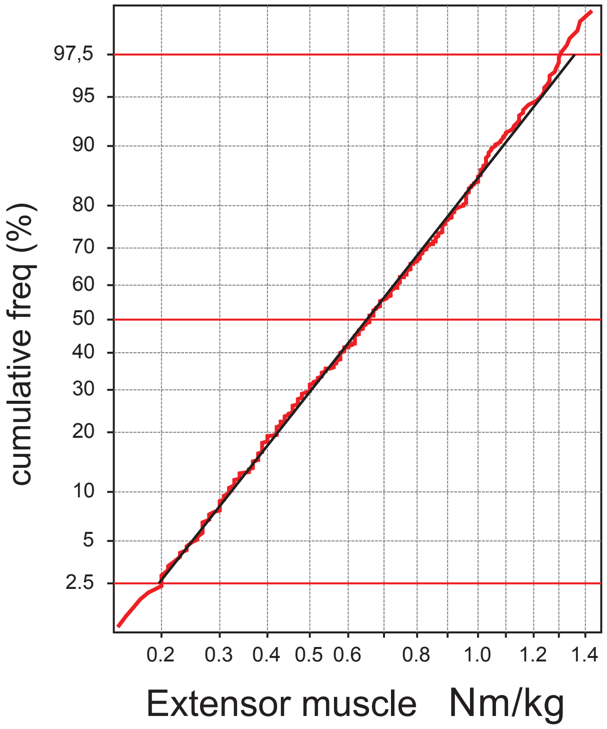

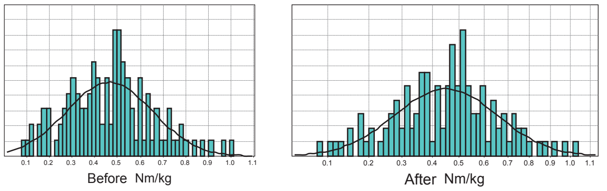

Flexor muscle strength(Nm/kg) Male n=121
Para: 0.141 ~ 0.450 ~ 0.880
Nonpara: 0.122 ~ 0.480 ~ 1.162
Pow=0.601 TPos=-0.013
Kurt=-0.251 Skew=-0.247
K-S test P=1.000 (N.S.)

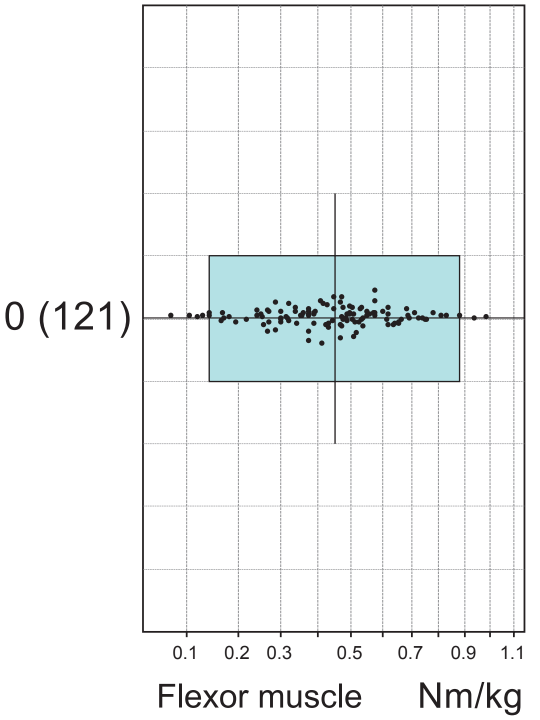

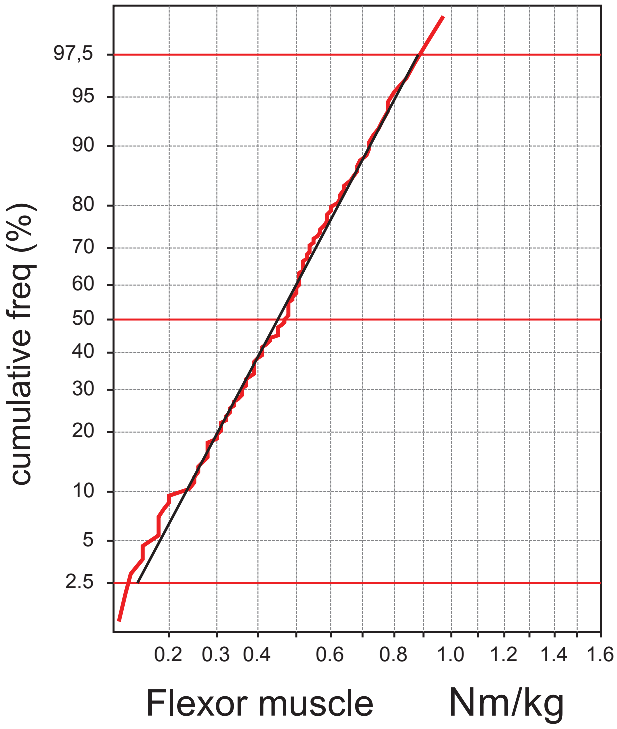

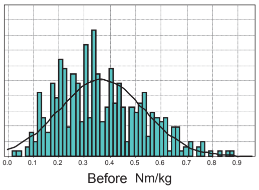

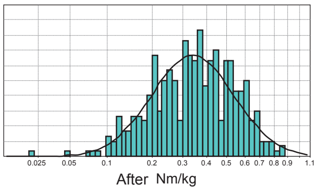

Flexor muscle strength(Nm/kg) Female n=403
Para: 0.110 ~ 0.338 ~ 0.791
Nonpara: 0.110 ~ 0.340 ~ 0.728
Pow=0.294 TPos=0.002
Kurt=-0.574 Skew=-0.192
K-S test P=0.332 (N.S.)

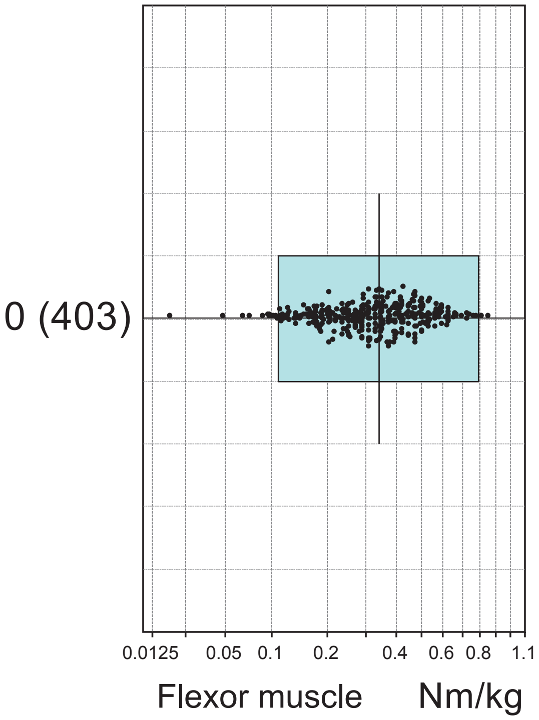

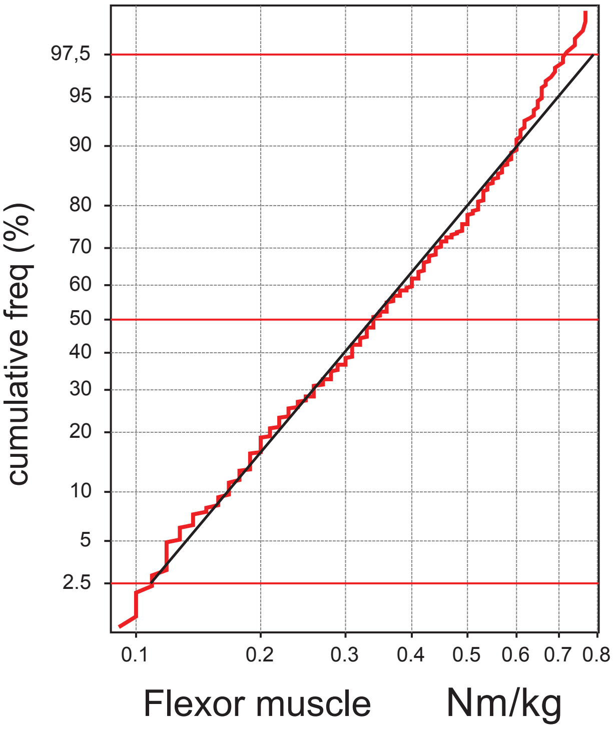

Supplement: S1 Fig — (PDF) [file pone.0249564.s001.pdf]
