# Supplementary material for: Determination of reference intervals for knee motor functions specific to patients undergoing knee arthroplasty
Source: PLoS One. 2021 Apr 14;16(4):e0249564. doi: 10.1371/journal.pone.0249564 (PMC8046200; doi:10.1371/journal.pone.0249564)
Supplement: Attachment — Submitted filename: Review of Study_PLoSOne_D-20-18248_for upload.docx [file pone.0249564.s003.docx]

Review of:
**Reference intervals of knee motor function tests for patients undergoing knee arthroplasty**

**Further Review Comments to the Author** The authors provide a technical investigation of normal recovery profiles in surgical interventions for KOA with respect to function (TUG) and impairment (strength and ROM) measurements that are feasible to measure in the clinic, and the associated effect of factors (age, gender etc) pre operatively and 14-days post operatively. Overall, I found the manuscript difficult to read and I think this is because the rationale was not entirely clear, and there is no clear mapping of objectives to the results and discussion sections. Based on this, I think the manuscript requires re-writing. I offer more detailed feedback below.

**Introduction & Rationale**

I sense the focus of the paper is in its utilisation of deploying statistical techniques to derive RIs that addressed the problem of non-normal data distributions. What I think is lacking in the introduction and discussion is a clear clinical rationale that this will help clinical practice. I agree that clinicians would welcome predicted recovery trajectories of typical KOA patients undergoing surgery based on the variables, but fear that these data alone might not affect practice due to the many other factors under consideration that the authors do not acknowledge e.g. fear of movement post-surgery, smoking and lifestyle choices. I could not see a clear statement in the introduction nor discussion that defends why *this* (albeit large) sample is definitively representative of the patient population in Japan. There is no information detailing the recruitment process for either group or justification for the discrepancy between the two groups sample size. The data do offer a basis on which studies could now be developed to explore why patients might deviate from a “normative” presurgical state and a “normative” recovery, and for that this paper is welcome. But I think the paper could benefit from a specific allusion to this and to how this relates or interacts with “healthy” age-matched adults.

The introduction states that conservative therapy of KOA is the treatment of choice – I would like to read what conservative therapy is in this context, and what exactly defines epicondylar deformity to inform local surgical decisions so international readers can make a judgement on their own practice. A more explicit description of the clinical indicators for TKA surgery would strengthen the manuscript.

The authors cite 2 previous pieces of work (Bade et al 2014 & Mizner et al 2005) to defend the use of knee ROM, knee flexion/extension isometric strength and TUG by stating “The prognosis after these surgeries depends on the pre-operative and short-term post-operative capability of physical and motor functions” (L62-63). These two references do not in my opinion constitute an adequate defence of this statement by themselves and I would expect a much more compelling argument to be put forward regarding these particular measures seeing as they are the main basis for the paper.

References are missing for the section (L54-62) “Among the 25.3 million patients with KOA, 8 million have symptomatic disease, which poses a large socio-economic problem in Japan. KOA features degeneration and attrition of joint structure that result in ossification of the cartilage and surrounding tissue, which restricts knee movements and eventually leads to pain and gait disturbance. Conservative therapy is the primary choice, but with the development of deformity in one or both epicondyles, either unicompartmental knee arthroplasty (UKA) or total knee arthroplasty (TKA) has been the therapeutic regimen of choice. Recently, a less invasive regimen called minimally invasive surgery TKA (MIS-TKA) has become popular as an alternative to conventional TKA (C-TKA)”.

The authors allow an entire paragraph (L67-78) listing previous TUG normative work based on *healthy* adults citing mean and SD values. Further explanation as to why this is specifically relevant to this study would be beneficial. I note though that the normative TUG meta-analysis work by Bohannan is missing from these data, and indeed from the whole paper (Bohannon, 2006). This is strange as not only did this work take various human ethnicities into account, it also included data from disparate TUG instructions – mainly to perform TUG at “comfortable” or “self-selected” pace or “quickly”. Despite these disparities in method the observed summary data was homogenous in Bohannon’s work.

L79 – if a sample distribution is Gaussian I agree that a sample 95%RI could be expressed as mean ± 1.96SD, but would a population estimate be more useful (i.e. mean ± 1.96SE) yielding a mean and 95%CI?

As an aside comment - I was interested to note that TUG undertaken at a comfortable and maximal tempo was included in the current study. The authors did not as far as I could see justify why this would be helpful and did not explain if the two variants in instructions were undertaken randomly. I think dealing with these two criticisms would benefit the manuscript. I am guessing the debate about maximum versus comfortable comes from the disparity in instruction language. According to some literature, for example (Kamide et al., 2011), the original TUG paper (Podsiadlo and Richardson, 1991) instructed participants to perform the TUG at a usual or comfortable pace, whereas a later protocol (Shumway-Cook et al., 2000) asked participants to perform TUG with maximum effort. This is an understandable but, in my opinion, incorrect interpretation of there being disparate protocols and I think alluding to this disparity is unhelpful.

This is because first, the Shumway-Cook paper asked participants to perform TUG “quickly and safely”, not “with maximal effort” nor “as fast as possible”. Second, I think Shumway-Cook and colleagues included a temporal qualifier (“quickly”) simply because participants were instructed to perform TUG under multiple dual-task conditions in their experiment. It is of course possible that within-subject TUG performance can be influenced by instructions to perform the task either comfortably or maximally, so there is a practical reason to include both protocol phenotypes in determining reference values as did the authors in the paper under review but only if there is a debate in the community about how to instruct the TUG. My interpretation is there is no such debate among clinicians. Even so, if a strong case could be constructed in this manuscript, then I would expect the two protocols to have been undertaken by an independent group or sufficiently apart and randomly by the same individuals to avoid any interaction effects. In contrast, it is interesting that forward average walking velocity was only measured at maximal tempo in the authors’ paper. Why not comfortable pace as well?? I would also like to see the authors defend the use of maximal walking velocity within the context of average self-selected walking velocity parameters (step width and length) being observed to be less variable than velocities under maximal volition (Sekiya et al., 1997).

Overall, I think the introduction lacks some clarity and would benefit from a more compelling argument for why this work is needed, and what it aimed to add for clinical practitioners or researchers.

**Method**

Patients – the sample size is large (n>500), which is an advantage, across 13 institutions which is a disadvantage without some description of differences in patients and clinical practice between them so readers can make an informed decision about the validity of the study. For example, while the inclusion criterion of indication for surgery is sensible it would be good to know if the different institutions deployed similar clinical reasoning in their decision making and whether patients were included on that decision or not.

The allusion from L107 that a standard clinical rehabilitation is shared across the institutions is welcome, but could be moved to be more prominent within the paragraph. Additionally, I would like to see a better breakdown of what the specification of the rehabilitation programme is here in addition to L109-112, either tabulated or in supplementary material, or signposted for the reader if it exists later in the manuscript. It needs to include dosing parameters and specification of any rehabilitation beyond simply practicing functional tasks.

There is no indication to when recruitment or data collection started, how long data were collected for, or how much missing data there were. I would expect these to be included for completeness. Why was the long-term follow-up of patient difficult? Were patients recruited affected by the COVID pandemic, did they have to self-isolate / were they deconditioned prior to surgery?

I would to see how many eligible participants did not consent to be included in the study for completeness. L99 – the exclusion of co-morbid neurological sensorimotor dysfunction – this I think needs to be more specific e.g. acquired focal neurologic, or chronic deteriorating neurologic condition affecting individuals’ ability to walk.

L100 – exclusion criterion 2) I think could be made clearer, and criterion 3) would be simpler described as cognitive impairment.

L115 – how were the healthy volunteers recruited? I would like to see more detail about the healthy participant inclusion criteria 1) and 2) for completeness.

L123 – the research design sentence could be made more succinct.

**Measurements**

I would expect to see the K-L classification referenced please.

L136 – while the same chair was used for TUG (in all institutions?), its height and whether it had arms would be useful information here for completeness. What was the go signal - Audible? Visual?

L141 – as per above, I am not convinced that Shumway-Cook specified “maximum” tempo in TUG. The MWS excluded acceleration and deceleration phases which is well explained. Like TUG, the go signal for MWS specifics are unclear and should be included, I think.

There is lack of detail within the functional measurement methods section to both justify the approach, allow repeatability, and defend any validity risks. An inclusion of this detail would be beneficial and strengthen the paper. For example, there is no allusion to the reliability or validity of the hand-held dynamometer technique and simply referencing the previous paper (Katoh and Yamasaki, 2009) is not enough. Can the authors account for the quality of the data they used? how many different researchers undertook the measurements? For the muscle testing it was unclear to me whether the ankle was fixed passively to a wall as shown in the Katoh paper, or did the researcher fix the tibial segment manually? A figure might help here. What verbal encouragement was offered to the participants? Was it standardised? What is the justification of taking the mean of 2 (and not more) knee extension muscle contraction attempts? Can the authors account for a first trial response in their data (Allum et al., 2011)? L169 – knee flexion strength is measured in the same manner as knee extension but there is not an allusion to the mean of two attempts for knee flexion strength. I would encourage consistency and clarity in the document. I am unconvinced that the torque calculations could be repeated as it is unclear where the measurement of the dynamometer was taken with reference to the knee joint – i.e. was it the top, bottom, centre-line, other part of the dynamometer?

The method for measuring knee ROM is extremely thin and should not rely on a reader having to read the reference supplied. It needs to be completely rewritten and expanded for completeness. There is no allusion to what landmarks the femur and tibial segments are designated by nor where the approximation of the knee joint centre is. There is also no allusion in the manuscript to reference positions i.e. is a straight leg 180 degrees extension or 0 degrees flexion? I would expect this detail to be at least included in the supplementary material.

**Statistical Methods**

I would like to see some justification about why the candidate factors were selected either by previous work, the authors’ experience, a logical argument, or other in the regression model. The factors selected seem sensible, but why these and not others? As it stands, I am not convinced why e.g. chronicity of OA, smoking, educational level, or living alone were not included as factors. The justification for selecting a rp≥0.2 simply because it is midway between the subjective small and medium correlation effect sizes offered by Cohen (Cohen, 1992) is by itself a weak justification in my opinion; I would prefer to see more justification of effect based on the context of the functional measures undertaken in this paper not just on a reported, yet essentially arbitrary, selection of numbers.

The rest of the statistical processing reported appears to be sound and referenced appropriately. I have one query as to why the 2 specific methods of transformation for MRA and SDR calculations were deployed for the TUG and strength data? In calculating the RIs, why was a Box-Cox transformation undertaken for all data without reporting on its normality distribution first? If there are any weaknesses or controversies in the statistical approaches used, then I would like to see them discussed, or at least acknowledged, in the discussion section.

The ethics paragraph is clearly written and welcome, it could be moved to the top of the methods section. Suggest change “We provided participants with a comprehensible document explaining this study…” (L234) to “We provided a written explanation of this study in language that was understandable to participants… “.

**Results**

L248 – I would like to know if the healthy control group were statistically different to the patient participants based on the observed demographics.

Notwithstanding the criticisms already noted, Table 1 and 2 are very clear and their captions thorough.

Figure 1 & 2 are a nice visual representation of these data. I note the data are presented transformed and wonder if the non-transformed data might be more meaningful to the reader and justify the use of the median and the inter-quartile range. Not sure why the ROM data are in histograms, the x-axis is already accounting for discreteness in the data.

L370 – “The RIs derived are listed in seven blocks in Table 3. Please note that RIs were derived by a parametric method for motor function tests except for extension and flexor ROMs, for which nonparametric methods were used.” I may have missed it, but the non-parametric version is opaque in the methods section.

Overall, I found the results section too long and suggest some editing to lighten the load on the reader.

**Discussion**

The first 2 paragraphs outlining the background is too long. I think it should simply remind the reader of the objectives, a brief justification of why this is important and/or unique, and perhaps the main findings.

L413 – “Factors associated with presurgical motor functions” section. This alludes to TUG but not which phenotype. Overall this section merely repeats the results which are not particularly compelling and offers no discussion point. As it is, I would remove.

In contrast L425 section offers more discussion, but I would remove repeating results with no discussion point.

L437 section alluding to healthy data – this should be a major discussion point, but as outlined above should reference the background where a defence of the need to present data of typical *patient* populations peri-operatively is required in order to quickly recognise outliers who might require more tailored management following their surgery..

L446 – “Final decision on partitioning”, and L475 “Reliability of the parametric method ..”section, again these sections could do with some editing and more discussion. It is difficult to clearly map the authors’ objectives for the paper with the results and discussion sections –there were too many surprises in these two sections I was not prepared for making it difficult to read.

L497 – “Clinical utility of RIs …” section. Sadly, this is the last, smallest, and rather meagre discussion section. Here I was desperate to see the authors *discuss* the RIs data. Instead this section lacks anything except that the RIs have been reported. This is a major omission in my opinion and completely under sells the main point of your work.

I would like to see much more discussion of the data and the fact that the authors could only follow-up patients 14 days post-surgery due to either lack of resources or because follow up care was too complex. This is alluded to in the limitations section, but why was this design undertaken if this limitation was known a priori?? The conclusion lacks any examples of how the RIs, despite their limitations, could be used objectively in practice by clinicians and researchers.

Finally, there were some trivial edits outlined below to take or leave.

L27-28 The description of the ‘objective peri-surgical management’ in the abstract would be better written as ‘crucial for determining the effectiveness of KA surgery’ or ‘crucial part of objective measures’

L50 ‘objective peri-surgical management of KOA’ might be better as ‘determining the effectiveness of KA surgery in patients with KOA.’

L55 ‘KOA features degeneration and attrition of joint structure that result in ossification of the cartilage and surrounding tissue, which restricts knee movements and eventually leads to pain and gait disturbance.’ might be better ‘KOA features degeneration and attrition of joint structure that result in ossification of the cartilage and surrounding tissue. This restricts knee range of movement causing pain and gait disturbance (expect a reference here).

L58-60 ‘Conservative therapy is the primary choice, but with the development of deformity in one or both epicondyles, either unicompartmental knee arthroplasty (UKA) or total knee arthroplasty (TKA) has been the therapeutic regimen of choice.’ Might be better ‘Conservative management is the primary treatment for KOA. However, a TKA / UKA is suggested if the patient fails conservative management’ - plus other clinical indictors previously discussed.

L63 ‘pre-operative’ would be better ‘pre-operative status’

L85 ‘With this background’ could be changed to ‘considering this’

L130 ‘A query was made on the status of regular exercise, and we regarded it positive when 30 minutes or more exercise was performed twice weekly or more.’ Would be better written as ‘Regular exercise was defined as exercise performed a minimum of twice weekly for 30 minutes or more’

L135 * L143 probably not use the acronyms in these titles and use full measurement names

L146 might read better as ‘signal’ rather than ‘sign’

L179 I think ‘a’ is missing before ‘multiple regression’, or alternatively plularise

L190 ‘As a factor for partitioning values in deriving RIs, we considered sex, age, BMI, and surgical mode as candidates.’ Might read better as ‘factors which may influence the RI’s include sex, age, BMI, and surgical mode.’

L241 regular exercise description is in the methods and is duplicated here, consider removing

L305-306 ‘It is obvious that muscle strength is generally lower in the women’ suggest change to ‘extensor muscle strength post-operatively is reduced in females’

L345 suggest change from ‘larger’ to ‘greater’

L350 suggest change ‘Because’ to ‘As’

L370 suggested change from ‘Please note that RIs were derived by a parametric method for motor function tests except for extension and flexor ROMs, for which nonparametric methods were used. To ‘It is important to note that RIs were derived by a parametric method for motor function tests except for extension and flexor ROMs where nonparametric methods were used instead.’

L394 suggested change from ‘and thus they take a long time to reach a favorable level’ to ‘and thus they take a long time to reach their optimum’

L394-395 change from ‘Therefore, it is important to detect patients who are lagging behind in the recovery process.’ to ‘Therefore, it is important to detect when patients are not aligning with expected recovering profiles in a timely manner.’

L398-402 is a long sentence to parse. Suggest breaking up.

L404 suggest change ‘ideal’ to ‘important’

L430 ‘was’ change to ‘were’

L438 and 441 change ‘suppressed’ to ‘reduced’

L447 suggested change from ‘In judging the need for partitioning of the test results, we relied on SDR.’ ‘we relied on SDR to judge the need for partitioning of the test results.’

L455 suggest change ‘but’ to new sentence ‘However,’

L460 suggested change from ‘There was a similar issue for’ to ‘There were’ and pluarise

L487 suggest change ‘obviously’ to ‘clearly’

L494 suggested change from ‘again’ to ‘furthermore or nevertheless’

L507 ‘data size’ suggested change to ‘the sample size’

L509-510 ‘For the long-term management of KOA patients who undergo knee arthroplasty, lower-limb motor function should be monitored for many months’ suggested change ‘As part of the long-term management of patients who undergo knee arthroplasty, lower-limb motor function scores should be monitored.’ Also suggest adding in *why* this would be beneficial would strengthen the manuscript.

L500-501 ‘To the best of our knowledge, there has been no such a report in the past.’ Suggest to remove as repeated in the conclusion

***6. PLOS authors have the option to publish the peer review history of their article (what does this mean?). If published, this will include your full peer review and any attached files.**

Yes

***Confidential to Editor**

I have no competing interests.

**References**

Allum, J.H., Tang, K.S., Carpenter, M.G., Oude Nijhuis, L.B., and Bloem, B.R. (2011). Review of first trial responses in balance control: influence of vestibular loss and Parkinson's disease. *Hum Mov Sci* **30**(2), 279-295.

Bohannon, R.W. (2006). Reference values for the timed up and go test: a descriptive meta-analysis. *J. Geriatr. Phys. Ther.* **29**(2), 64-68.

Cohen, J. (1992). A power primer. *Psychol. Bull.* **112**(1), 155-159.

Kamide, N., Takahashi, K., and Shiba, Y. (2011). Reference values for the Timed Up and Go test in healthy Japanese elderly people: determination using the methodology of meta-analysis. *Geriatr Gerontol Int* **11**(4), 445-451.

Katoh, M., and Yamasaki, H. (2009). Comparison of Reliability of Isometric Leg Muscle Strength Measurements Made Using a Hand-Held Dynamometer with and without a Restraining Belt. *J Phys Ther Sci* **21**(1), 37-42.

Podsiadlo, D., and Richardson, S. (1991). The timed "Up & Go": a test of basic functional mobility for frail elderly persons. *J. Am. Geriatr. Soc.* **39**(2), 142-148.

Sekiya, N., Nagasaki, H., Ito, H., and Furuna, T. (1997). Optimal walking in terms of variability in step length. *J. Orthop. Sports Phys. Ther.* **26**(5), 266-272.

Shumway-Cook, A., Brauer, S., and Woollacott, M. (2000). Predicting the probability for falls in community-dwelling older adults using the Timed Up & Go Test. *Phys. Ther.* **80**(9), 896-903.
