# Supplementary material for: Determination of reference intervals for knee motor functions specific to patients undergoing knee arthroplasty
Source: PLoS One. 2021 Apr 14;16(4):e0249564. doi: 10.1371/journal.pone.0249564 (PMC8046200; doi:10.1371/journal.pone.0249564)
Supplement: Attachment — Submitted filename: Responses to Reviewers comments towards R2 20210215.docx [file pone.0249564.s005.docx]

**Reviewer #1**

Our response → We greatly appreciate your review of our manuscript and the invaluable comments provided to improve it. We addressed each issue and revised the manuscript accordingly as described below.

1. Line 226: misspelled name „Dbson” instead of Dobson

2. Lines 258-260: description of tibia and femur axis seem to be swapped; description for tibia fits to femur and reversely description for femur fits to tibia.

3. Table 1, row about sex: plural “men” should be written instead of singular “man”.

4. Lines 396-400: First, an information about extensor strength reduction in women is given. Next, it is repeated for both sexes, which is true, but there is no need to state it twice.

Our response →We corrected the errors as suggested.

5. Line 516: strictly speaking, no data from Table 4 directly prove that distribution was not Gaussian before transform.

Our response → We thank you for pointing out this mistake. We removed the following phrase from Table 4: “*the distribution of TUG values is not Gaussian as is evident from our* ***~~Table. 4~~*** *~~and~~* ***Suppl Fig. 1***”

**Reviewer #2**

- Although the authors made most of the suggestions, the objectives, results presented and discussions are still not clear for the reader to follow a clinical reason.
I suggest that the authors write the clinical meaning for each result found in the discussion.

Our response → We are grateful for your review of our manuscript again. We realized the problem of describing our objectives of this study comprehensibly in the Introduction and the lack of our statement of clinical implications of this study in the Discussion. We concisely described the objectives as follow:

“*With these backgrounds, we conducted a multicenter study to evaluate sources of variation of knee motor functions and to establish their RIs specific for KOA patients undergoing KA.*

*We aimed at using the quantitative information for objective implementation of peri-surgical rehabilitation and for screening patients who are deviated from the normal course of recovery*.”

We added the clinical implication of this study as follows at the end of the Discussion:

“*In summary, a clinical implication of this study is as follows. Since we only enrolled patients who followed the normal course of recovery, the quantitative information of RIs and their factors of variations obtained in this study will facilitate peri-surgical rehabilitation customized to patients according to sex, age, and surgical mode. Furthermore, the subgroup specific RIs will be useful in detecting patients who are deviated from the normal course of recovery.*”

**Reviewer #3**

The authors have improved the manuscript based on all reviewers’ comments. The rationale is clearer and the statistical techniques are better described now

Overall, I still find the manuscript difficult to read and I contend it could be made clearer and simpler with better mapping of the stated objectives to the results.

Our response → We are grateful for your time and efforts to review our manuscript again in great detail to give us a chance to improve it.

First of all, we realized that the following three descriptions of our objectives in the Introduction were still incomprehensible and misleading and thus caused further confusion.

“With these backgrounds, we conducted this study as a team of physical therapists to provide optimal peri-surgical rehabilitation to KOA patients undergoing knee arthroplasty in collaboration with orthopedicians. Our specific objectives were:

(1) For objective use of knee motor function tests in the rehabilitation, to explore their sources of variation (SVs) and to determine their reference interval (RIs), which are specific to KOA patients undergoing knee arthroplasty.

(2) To apply up-to-date statistical methods in analyzing the SVs and in determining RIs, which have not been used in the field of rehabilitation medicine in the past,

(3) To compare knee motor functions before and two-weeks after knee arthroplasty, which is of relevance in predicting a long-term success of the surgery. “

We think that this problem occurred due to an insufficient description of the role of physical therapists in the post-surgical rehabilitation of patients undergoing knee arthroplasty (KA). Therefore, we added the following description of how the peri-surgical rehabilitation is generally performed and how the knee function tests are being used by adding a new Figure 1.

“In the clinical management of KOA patients undergoing knee arthroplasty, physical therapists take an important role in providing peri-surgical rehabilitation in close collaboration with orthopedic surgeons*. The scheme of rehabilitation according to a common clinical pathway for knee arthroplasty is as shown in* ***Fig. 1****. The functional status of patients is usually assessed by performing knee motor function tests of a safe variety, such as timed-up-and-go (TUG) test, maximum walking speed (MWS), knee muscle strength, and knee range of motion (ROM) [4].*

*These test results are often referred to in deciding a patient’s discharge date.* *To read the results objectively, it is essential to understand their sources of variation and to have a reliable reference interval (RI) for each parameter.”*

Therefore, we adopted only the first objective and deleted other two because of their misleading nature and irrelevancy for this study.

The first objective is now re-written as follows at the end of the Introduction:

“*On the basis of this background, we conducted a multicenter study to evaluate sources of variation of knee motor functions and to establish specific RIs for KOA patients undergoing KA. We aimed to use quantitative information for objective implementation of peri-surgical rehabilitation and for screening of patients who deviate from the normal course of recovery.*”

As for objective (2) of applying up-to-date statistical methods, we now regard it not so important and thus we toned down this point and substantially reduced discussion on the past “normative value” studies.

For objective (3) of comparing knee functions before and after KA, we found the description was misleading because immediate post-surgical test results from our study cannot be used in predicting long-term patient outcome, although there are some studies that claimed the importance of immediate postsurgical knee functions in relation to long term outcome.

I think the authors want to say that previous reporting of RIs in predominantly healthy subjects are flawed statistically and offer methods as an alternative approach which is a valid purpose. Reporting RIs in healthy subjects using the alternative statistical technique might be the sole purpose of a separate paper.

Our response → As we just mentioned above, we found it irrelevant to cite past studies of knee motor functions based on healthy elderly subjects, regardless of the statistical methods used. Therefore, we deleted some of those description in the Introduction and the Discussion. Thus, we have no plans to write a companion paper to this one because the two populations (healthy vs. KOA) are too mismatched to make any worthy comparison.

They then suggest that the reporting of specific motor functions (TUG, max walking velocity, leg ROM, & specified lower limb strength) is relevant.

I am still to be convinced by the authors’ manuscript that they recognise that these measures are examples of proxies of physical functional constructs and that there exist other measures – so why these specific ones and not others, and why measured this way??

Again, this debate might provide a separate manuscript worthy of publication.

Our response → We understand that there are many other measures of knee motor functions, such as the 6-minute walking distance and a stair climb test. However, during the immediate post-surgical period that physical therapists are involved in, these tests are generally not performed for the sake of safety. Unfortunately, a second study targeting other physical function tests is beyond the scope of our routine clinical practice.

Regardless of this criticism, they go on to provide RI data for these measures which is in itself sensible and useful for discriminative use in clinical practice or research given the statistical flaws described in previous work.

Our response → We toned down the issue of statistical flaws in the past normative value studies in the Discussion because of the irrelevance of the issue based on the main objectives of this study.

They content that these data are useful in clinical Japanese practice as a means to determine if a patient is starting from a deranged functional profile pre-surgery, or is deviating from a normal recovery trajectory after surgery based on the functional measures selected.

But, this reader is not convinced the authors have justified that their sample represents a typical patient sample.

This is mainly because they openly admit they were limited in their design to determine whether their sample reached a pre-determined threshold of functional recovery longitudinally i.e. whether the longer-term outcome of their surgery was successful.

We simply do not know if this is the case, and therefore there is doubt their RIs represent a compelling acceptable range. This needs to be better acknowledged in my opinion.

Our response → We again apologize for having cited misleading papers in the Discussion, which reported the importance of short-term post-surgical motor functions in predicting the long-term recovery of patients. We admit that we cannot say anything about the long-term success of the surgery.

As for the implication of the RIs we derived, we had missed important descriptions on how we enrolled KOA patients in the Methods. We just included patients who experienced a normal recovery course and were discharged within 3 to 4 weeks after KA. Therefore, our RIs can be referred to in judging whether a given patient is in a normal recovery course for scheduling discharge. We also need to emphasize that the clinical pathway we adopted in this study is widely used in Japan, and thus the results of this study are relevant in the clinical practise of peri-surgical rehabilitation of KA patients in Japan.

Since we clarified the sources of variation of the test results, an additional clinical implication of this study is that it was made possible to interpret test results more appropriately in considerations of sex, age, and surgical mode.

In the revised manuscript, we added these points as a clinical implication of this study in the last section of the Discussion:

“*In summary, the clinical implication of this study is as follows. As we only enrolled patients who followed a normal course of recovery, the quantitative information of RIs and their factors of variation obtained in this study will facilitate peri-surgical rehabilitation customized to patients according to sex, age, and surgical mode. Furthermore, the subgroup-specific RIs will be useful in detecting patients who deviate from the normal course of recovery.*”

All in all, I think the authors have a made a decent attempt effort to incorporate and/or rebut the 3 reviewers’ comments. While the manuscript is improved, it still requires some revision.

It could benefit from breaking into clear sections or even be incorporated into two manuscripts in my opinion.

Our response → We hope we have clarified our objective and clinical implications of this study as described above in responding to your invaluable suggestions one by one. We also hope that it will not be necessary to write other related manuscripts, which seems not possible from the objective of this study.

**Specific points and minor Edits** (line numbers refer to tracked-changed manuscript version):

L82 onwards - Explain what the differences between the different surgical types in the introduction after L82.

Our response → We added the following description of the surgical modes:

“Either unicompartmental knee arthroplasty (UKA) or total knee arthroplasty (TKA) has been the therapeutic regimen of choice. *The former is a less invasive technique that only replaces a single granular area and preserves the anterior and posterior cruciate ligaments* [2,3]. Recently, as an alternative to conventional TKA (C-TKA), a minimally invasive TKA surgery (MIS-TKA) has become popular*, which features a shorter skin incision than that with C-TKA*.”

L43 -Change ‘as controls’ to ‘a control group of 120 healthy elderly volunteers’

L72 - Change ‘by Yoshimura et al.’ to ‘Yoshimura et al. (2011) reported in a large cohort study the prevalence of knee osteoarthritis (KOA) is as high as 42.6% in men and 62.4% in women over 40 years old across Japan.

L85-88 - Change ‘For proper management and smooth recovery through rehabilitation, it is important to monitor knee motor functions [4] such as timed-up-and-go (TUG) test, maximum walking speed (MWS), knee muscle strength, and knee range of motion (ROM).’ To ‘It is important to use objective tests such as timed-up-and-go (TUG) test, maximum walking speed (MWS), knee muscle strength, and knee range of motion (ROM) to monitoring performance post operatively [4].’

L113 - Change objective 1 to ‘to explore sources of variation (SVs) and determine their reference interval (RIs) of objective tests used to measure knee motor function within KOA patients undergoing knee arthroplasty’

L116 - Change objective 2 ‘To be the first study to explore statistical methods for analysing the SVs and determining the RIs.’

Our response →We revised the wording as suggested.

L118 -Change objective 3 to ‘To compare knee motor functions before and two-weeks after knee arthroplasty as a predictor of long-term success of KA surgery.’

Our response →We deleted the misleading objective 3 as explained above.

L124 - Change ‘A total of 583 KOA patients undergoing elective knee arthroplasty were recruited consecutively between July 2013 and February 2018 by use of harmonized study protocol from 13 institutions specialized in knee arthroplasty, which are scattered widely in western Japan.’ To ‘A total of 583 KOA patients undergoing elective knee arthroplasty were recruited from 13 institutions across Western Japan that specialized in knee arthroplasty between July 2013 and February 2018’.

Our response →We corrected the description as suggested. Please note that in the revised manuscript, we stated that we originally recruited 623 KOA patients, but we excluded 41 subjects who did not follow a normal course of recovery as well as 38 subjects with missing test results. Therefore, after the above revised description, we added the following facts about the enrolled cases.

“*After exclusion of 38 patients with missing motor function test results and 41 patients who had a delay in discharge, a total of 545 patients were regarded as eligible for the subsequent analyses.*”

L140-149 – The objectives are clearer now. But similar to previous criticisms of a lack of a clear mapping of objectives to the data collected - I do not see why a group of healthy subjects was recruited with these objectives in mind – the justification was not made clear to me in the methods section either. I had to read ahead to L395 in the results for a first mention of how the healthy data was used and why.

Our response → As mentioned above, we admitted the irrelevance of knee motor functions of healthy subjects, and we just described past normative value studies briefly. Nevertheless, to show the needs of the RIs specific for KOA patients, we think it necessary to show the test results of age-matched healthy subjects in this study. Therefore, we hope the healthy subjects’ results can be retained in the Results to emphasize the importance of deriving “disease-specific” RIs.

L161 – Define DM in full if using; but surely peripheral neuropathy of any aetiology would be the exclusion criteria not only diabetes?

Our response → We revised the description of the excluded conditions as follows:

“1) presence of motor paralysis or other neurological dysfunctions such as stroke, *lumbar disc herniation, spinal canal stenosis, peripheral neuropathy of diabetes mellitus* unrelated to KOA,…”

L165 – “… enrolled were 545 subjects.” Suggest change to “…545 subjects were enrolled.”

L173 – Change “followings” to “following”

L211 – Change “… KOA patients …” to “… KOA patient …”

L214- Suggest change “… on a chair …” to “… on an armless chair with seat-height set between 40 and 40cm high …” and delete the next sentence.

L258-260 – I think the descriptors of the tibial and femoral segments need changing around.

L275 – “… in P value …” is better as “ … as a P value …”

L277 – pluralise “… coefficient …” I think

L317 – suggest change “… we applied nonparametric …” to “… we applied a nonparametric …”

L339 – “… Mann-Whiney…” should be “… Mann-Whitney …”

L340 – I think pluralise the word “test” when it appears in this sentence, and these analyses should be in the methods section.

Our response →We thank you for these suggestions, which we applied to the manuscript.

L340-342 - Allusion to, or interpretations of, “small” or any statistical differences should be included in the discussion section – the results should merely report the results I think

Our response →According to the suggestion, we rephrased the results as follows removing the interpretations: “*Statistically significant differences were found for higher age and BMI, and less frequent habit of exercise observed among KOA patients.*”

L408 – Table 3, there is no adjacent mention of Table 3 in the text here, I had to look to L424 in the next section to find it, Table 3 needs better placement in the text

L506 – suggest change “… proper …” to “ … optimal …” or similar

L580 – I am not sure you need “on the other hand ….” At the start of the sentence here

Our response → We thank you for these suggestions, which we accordingly applied to the manuscript.
