# Supplementary material for: Determination of reference intervals for knee motor functions specific to patients undergoing knee arthroplasty
Source: PLoS One. 2021 Apr 14;16(4):e0249564. doi: 10.1371/journal.pone.0249564 (PMC8046200; doi:10.1371/journal.pone.0249564)
Supplement: Attachment — Submitted filename: Responses to the Reviewers comments towards R3.docx [file pone.0249564.s006.docx]

Reviewer #1:

Previous comments has been addressed and manuscript is in general sound.
Some minor changes, listed below, are still needed, but they do not affect the content substantially. Line numbers below refer to the text with highlighted changes.
Lines 355-363: The authors probably meant Fig 3, not 2, here.
Line 435: There should be Fig 4, not 3.

Our response → We thanks again for the review of our revised manuscript and the provision of the comment on the problem of incorrect numbering of Figures. We corrected them accordingly.

Reviewer #3:

The authors have made an excellent attempt to consider all the reviewer’s comments and there is no doubt this is an improved manuscript. The manuscript provides RI data for the functional measures in a real set of patients which is sensible and of use to the community for discriminative use in clinical practice or research given the statistical flaws described in previous work. They contend that these data are useful in clinical Japanese practice as a means to determine if a patient is starting from a deranged functional profile pre-surgery, or is deviating from a normal recovery trajectory after surgery based on the functional measures selected.

Our response → We are grateful for your time and efforts to review our manuscript again and the kind offering to us of additional comments to further improve the manuscript.

Overall, I still find the manuscript difficult to read. Suggest to include in line 88 in the introduction what the sources of variation are/might be to prime the reader to the factors explored in the paper.

Our response → According to your suggestion, we added the following explanation for the term of “sources of variation” at line 88: “To read the results objectively, it is essential to understand their sources of variation *(factors that may alter test results without pathological conditions)* and to have a reliable reference interval (RI) for each parameter.”

I still think it could be made clearer and simpler with better mapping of the stated objectives to the results – e.g. crafting the manuscript so the objective of identifying factors associated with motor function parameters is clearly stated at the end of the introduction and in the methods, and a clear rationale why healthy subjects were included (again at the end of the intro and in the methods section from line 144), and some allusion to discussing the healthy data in the discussion section.

Our response → According to your advice, we also added the meaning of analyzing the sources of variation (SV) as follow **near the end of the Introduction**: “On the basis of this background, we conducted a multicenter study to evaluate sources of variation (SVs) of knee motor functions and to establish *well-stratified* RIs *in consideration of the SVs* for KOA patients undergoing knee arthroplasty.”.

Furthermore, the objective of stratifying the RIs in consideration of SVs had been mapped **in the Results** under the subheading of “**Assessment of factors to derive RIs specific to subgroups**” (just before the subheading of “**Determination of RIs**”) in describing the SV-based stratification of the RIs for the KOA patients.

Regarding the implication of recruiting healthy subjects and determining their RIs for the knee motor functions tests, we also added the following description **at the very end of the Introduction** to clarify the point according to your suggestion: “*Age matched healthy subjects were also recruited to ascertain the need for disease -specific RIs.* We aimed to use *these* quantitative information for objective implementation of peri-surgical rehabilitation and for screening of patients who deviate from the normal course of recovery.”

For this issue of our deliberate inclusion of age-matched healthy elderly, we also gave additional explanation of including them **in the Methods** under the sub-heading of “**Healthy volunteers**” as follows: “*As described in the Introduction*, we recruited 120 apparently healthy volunteers with comparable ages *to make a contrast of their knee motor functions with those of KOA patients undergoing knee arthroplasty*. They comprised 36 men and 84 women ……”

As for the need in the Discussion of additional description for the implication of deriving RIs from the healthy individuals, we had already described it as follows **at the third paragraph of the Discussion**: “In addition to these methodological issues of the past studies targeting healthy subjects, as demonstrated in Fig. 3 and 4, healthy ranges of knee motor function tests were quite different from those of KOA patients both before and after the surgery.…”
